# TRAIL and Cardiovascular Disease—A Risk Factor or Risk Marker: A Systematic Review

**DOI:** 10.3390/jcm10061252

**Published:** 2021-03-18

**Authors:** Katarzyna Kakareko, Alicja Rydzewska-Rosołowska, Edyta Zbroch, Tomasz Hryszko

**Affiliations:** 1 2nd Department of Nephrology and Hypertension with Dialysis Unit, Medical University of Białystok, 15-276 Białystok, Poland; alicja.rosolowska@umb.edu.pl (A.R.-R.); tomasz.hryszko@umb.edu.pl (T.H.); 2 Department of Internal Medicine and Hypertension, Medical University of Białystok, 15-276 Białystok, Poland; edyta.zbroch@umb.edu.pl

**Keywords:** TRAIL, cardiovascular diseases, cardiovascular risk, cerebrovascular disorders, apoptosis

## Abstract

Tumor necrosis factor-related apoptosis-inducing ligand (TRAIL) is a pro-apoptotic protein showing broad biological functions. Data from animal studies indicate that TRAIL may possibly contribute to the pathophysiology of cardiomyopathy, atherosclerosis, ischemic stroke and abdominal aortic aneurysm. It has been also suggested that TRAIL might be useful in cardiovascular risk stratification. This systematic review aimed to evaluate whether TRAIL is a risk factor or risk marker in cardiovascular diseases (CVDs) focusing on major adverse cardiovascular events. Two databases (PubMed and Cochrane Library) were searched until December 2020 without a year limit in accordance to the PRISMA guidelines. A total of 63 eligible original studies were identified and included in our systematic review. Studies suggest an important role of TRAIL in disorders such as heart failure, myocardial infarction, atrial fibrillation, ischemic stroke, peripheral artery disease, and pulmonary and gestational hypertension. Most evidence associates reduced TRAIL levels and increased TRAIL-R2 concentration with all-cause mortality in patients with CVDs. It is, however, unclear whether low TRAIL levels should be considered as a risk factor rather than a risk marker of CVDs. Further studies are needed to better define the association of TRAIL with cardiovascular diseases.

## 1. Introduction

The Greek term “apoptosis” meaning “falling leaves in autumn” was first used for the description of programmed cell death in 1972 by John Kerr [1]. Its origin dates back to Hippocrates [2], and since then, substantial progress has been made in our understanding of this naturally occurring process. Apoptosis can be triggered by various factors (biological, chemical, physical) and it is tightly regulated by a family of proteases called caspases, which are activated during cellular response to a specific trigger. Apoptotic signaling can be internal (intrinsic apoptosis) or external (extrinsic apoptosis). The intrinsic pathway can be induced by cellular stress or DNA damage. The extrinsic pathway is induced by signals activating cell-surface death-receptors. Apart from an important role in physiological processes such as aging, apoptosis (especially when it is inadequate) contributes to the development of many disorders including malignancies [3], and neurological [4] and cardiovascular diseases (CVDs) [5]. In recent years, a large number of studies have tried to clarify how this complex process is actively driven by the cells. One of the proteins involved in inducing apoptosis is TNF-related apoptosis-inducing ligand (TRAIL). TRAIL, which is also known as Apo-2 ligand (Apo-2L) or TNF super family 10 (TNFSN10), is a 281-amino acid type II transmembrane protein [6]. The membrane-bound TRAIL can be cleaved by cysteine proteases, and secreted into circulation, leading to the formation of the soluble form of TRAIL. Activated monocytes and neutrophils are the most important source of soluble TRAIL [7]. Interest in TRAIL rapidly increased when it turned out that TRAIL could selectively kill cancer cells whilst sparing normal cells [8]. This potential anti-tumor effect led to identifying TRAIL as a promising anti-cancer agent [9]. Those varied biological functions result from different receptors being activated by TRAIL.

**Figure 1:**
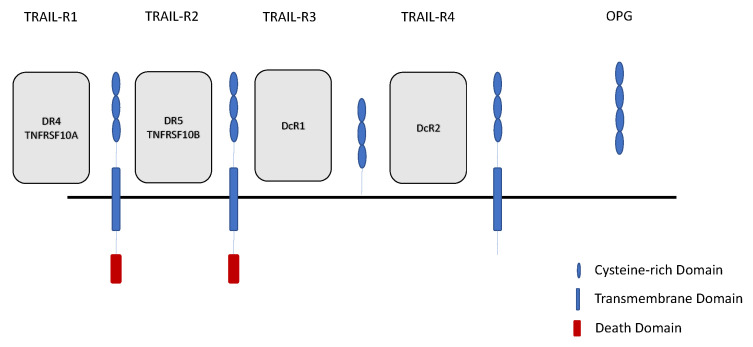
TRAIL receptors and their alternative names. TRAIL-R1 is also known as DR4 (death receptor 4) and TNFRSF10A (tumor necrosis factor receptor superfamily member 10A); TRAIL-R2 is also known as DR5 (death receptor 5) and TNFRSF10B (tumor necrosis factor receptor superfamily member 10B); TRAIL-R3 is also known as DcR1 (decoy receptor 1), TRAIL-R4 is also known as DcR2 (decoy receptor 2); OPG—osteoprotegerin (soluble decoy receptor).

Five TRAIL receptors have been identified: TRAIL-R1 and TRAIL-R2 (death receptors), TRAIL-R3 and TRAIL-R4 (decoy receptors), and osteoprotegerin (OPG, which is a soluble decoy receptor). Their alternative names are detailed in Figure 1. Decoy receptors act as molecular traps for agonists, binding TRAIL and keeping it from binding with death receptors. TRAIL-R1 and TRAIL-R2 have intracellular death domain motifs while TRAIL-R3 and TRAIL-R4 lack the cytoplasmic domains necessary to transduce apoptotic signal (Figure 1). Thus, TRAIL may exert pleiotropic effects, inducing apoptosis when binding to death receptors, but with TRAIL-induced apoptosis blocked by the decoy receptors. Soluble forms of those receptors are also found in plasma. TRAIL and its receptor system therefore seem to maintain a balance between apoptotic and antiapoptotic actions.

Recently, a lot of attention has been focused on the components of OPG/TRAIL axis as proteins implicated in the development of CVDs. The TRAIL receptors are expressed in the cardiovascular (CV) system in vascular smooth muscle cells [10] and cardiomyocytes [11]. Data from animal studies shows that TRAIL may possibly contribute to the pathophysiology of cardiomyopathy [12,13], atherosclerosis [14,15], ischemic stroke [16], pulmonary hypertension [17] and abdominal aortic aneurysm [18]. It has also been suggested that TRAIL might be potentially useful in CV risk stratification [19].

The association between TRAIL and CVDs is not well understood. We aimed to answer the question of whether TRAIL is a risk factor or a risk marker in CVDs, particularly taking into consideration major adverse CV events defined as a composite endpoint: nonfatal myocardial infarction, nonfatal stroke and CV death. We analyzed clinical and experimental data from human studies aiming to gather full knowledge on the subject in question.

## 2. Materials and Methods

The report of the methods used for this systematic review was in accordance with the Preferred Reporting Items for Systematic Reviews and Meta-Analyses (PRISMA) consensus statement. We conducted a comprehensive literature search in the electronic databases including PubMed and Cochrane Library up to December 29, 2020. Search strategies included keywords and Medical Subject Heading (MeSH) terms with all subheadings included. The MeSH terms were “cardiovascular diseases”, “cerebrovascular disorders” and “pulmonary embolus” (searched together using the Boolean operator “OR”). The keyword used were: “TRAIL” and “cardiovascular risk”. The terms were combined using the Boolean operator “AND”. We also searched the combined mode of MeSH or keywords. The results were merged with duplicates discarded. Further studies were sought by manually searching reference lists of the relevant articles. Relevant articles were selected based on their title, abstract or full text. Articles were excluded if they were clearly related to another subject matter or were not published in English, French, or German.

## 3. Results

**Figure 2:**
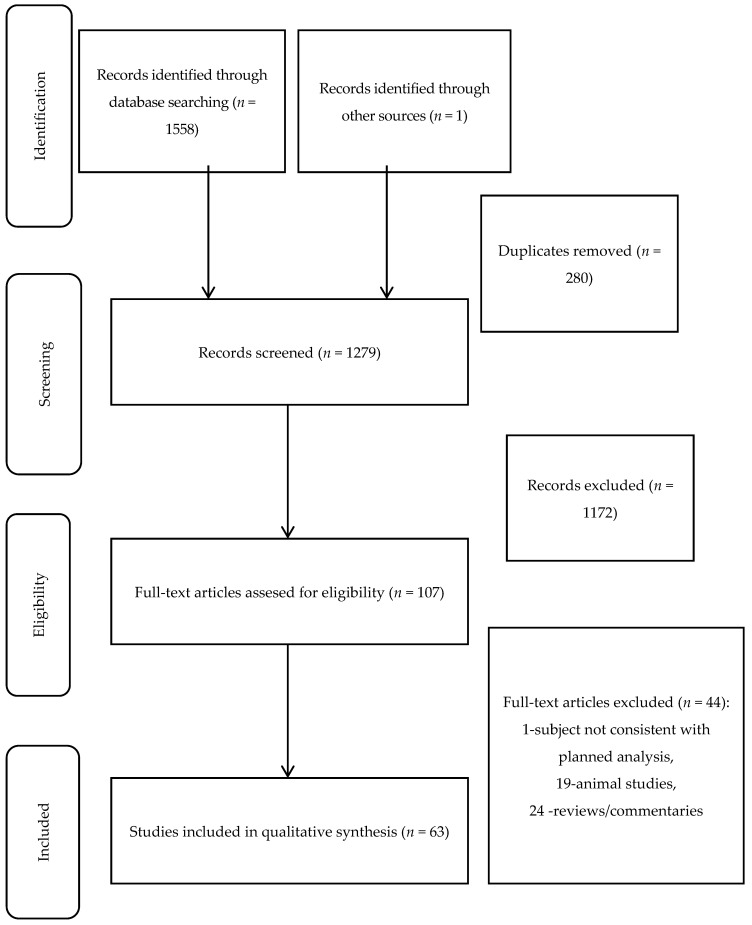
Search strategy and results.

The detailed process of literature searching is illustrated by flowchart (Figure 2). Initially, 1559 articles were identified from electronic databases, 1279 were screened, and 107 were retrieved and assessed for eligibility. Finally, 63 original studies were included in our systematic review. We have identified 48 clinical and 15 basic studies. All of them are detailed in Table 1, Table 2 and Table 3. Fifteen of those papers reported cardiovascular event endpoints. Due to their heterogeneity (different endpoints and measured proteins), the risk of bias was not assessed. We decided to summarize them together since they all included patient-related outcome measures (Table 3). From those 15 studies, nine were regarding TRAIL and six regarding TRAIL-R2. TRAIL was inversely correlated with all-cause death in patients with CVD in seven out of nine studies (one found an association that was not statistically significant); cardiovascular death was inversely correlated with TRAIL only in two out of three studies. In four out of six studies, higher TRAIL-R2 levels predicted CV death or a CV event.

## 4. Discussion

The intention of this review was to summarize current knowledge about the involvement of the TRAIL pathway in the development and prognosis of CVDs. Moreover, we tried to evaluate published studies focusing on cardiovascular outcomes. Unfortunately, we were unable to assess the quality of data on TRAIL and major adverse CV events because of the heterogenicity of the groups. Conducted studies had various endpoints and the number of studies with a similar endpoint assessing CVD risk was insufficient for our objective. We decided, therefore, to include all studies since we think the information gathered from them is very interesting and shows new clinical data in terms of TRAIL receptors and their prognostic utility.

The most important finding is that prospective studies demonstrated that lower TRAIL concentration predicted a poor prognosis in patients with CVD, e.g., Volpato et al. [69], Osmancik et al. [70], Richter et al. [72]. In the study by Volpato et al., a large cohort of patients with pre-existing CVD was evaluated over a period of six years. The authors demonstrated that baseline TRAIL concentration was inversely and independently related to all-cause and CV mortality. Participants with the lowest levels of TRAIL (the first and second quartiles) had a three-fold increased risk of CV and all-cause death compared to those with the highest TRAIL levels [69]. Similar observations were made previously in smaller cohorts of patients with heart failure [66] and acute myocardial infarction [67]. In the aforementioned study by Volpato et al., those conclusions were extended to patients with a wider range of CV conditions. The authors, based on their analysis, even suggested the usefulness of TRAIL for predicting outcomes in patients with subclinical CV conditions.

The theories behind the protective effect of TRAIL on mortality risk in CVD are various depending on the etiology of the CV condition, which are included in the discussion below. The mechanism of TRAIL’s beneficial effect might be exerted either by a direct impact on cells or indirectly by modulating systemic immune functions. In vivo (rodent models) studies showed that recombinant TRAIL injections contributed to a slower progression of atherosclerosis and plaque stabilization mainly by the induction of the apoptosis of infiltrating macrophages [81]. TRAIL recruits activated leukocytes to particular tissue and initiates apoptosis to terminate the immune response. On the other hand, it is speculated that TRAIL exerts its protective role not by stimulating the extrinsic apoptotic pathway but by activation of the survival/proliferation pathways [82], as is the case in HF patients [83].

At the same time, when low levels of TRAIL are determining the survival rate, TRAIL-R2 concentration is reported also as having predictive value but with an inverse relationship [74]. The soluble form of TRAIL death receptor was identified as a prognostic marker in CVD by proteomic studies [74,79,80]. During three-years of follow-up, a high concentration of TRAIL-R2 independently predicted future CV events in patients with advanced atherosclerosis, even after adjusting for traditional CV risk factors [52]. A cross-sectional study in patients with lupus erythematosus showed that patients with a range of CVDs had markedly higher TRAIL-R2 concentration than those without [51]. Mattison et al. performed a genome-wide association study to determine the role of death receptor-activated apoptosis in CVD and investigated whether genetic variants of death receptors were linked to CVD risk [75]. Identification of several single-nucleotide polymorphisms (SNPs) associated with the level of plasma soluble form of death receptors remained significant even when the impact of other metabolic factors was considered. Genetic factors play a role in determining the concentrations of soluble death receptors in circulation. A weak association was identified between SNP in the gene region for TRAIL-R2 and coronary artery disease, but as the authors concluded, it is unlikely that soluble death receptors alone play a functional role in CVD [75]. It is probably the whole process of apoptosis that increases the risk.

Interestingly, in two studies, TRAIL, apart from showing a correlation with all-cause mortality, was correlated also with death from infectious causes [68,71]. That observation might be a result of the possible role of TRAIL and its death receptors in defending organisms from viral and bacterial infections. In experimental models, strong up-regulation of the TRAIL apoptotic pathway was observed in response to infection. As a consequence, virus- or bacteria-infected cells were eliminated efficiently by TRAIL-induced apoptosis [84,85]. These results from animal data correspond with reports showing an inverse TRAIL relation with mortality in patients with sepsis [86].

A lot of data come from the population of patients with chronic kidney disease (CKD) and its most pronounced manifestation—end-stage kidney disease. These patients have a high mortality rate and the main cause of death in this group is CVD. Apart from accelerated atherosclerosis, the CKD population is characterized by an inadequate inflammatory response. The investigations on the potential involvement of TRAIL, since it modulates both processes, are therefore highly interesting. Liabeuf at al. found that TRAIL was inversely associated with mortality risk in CKD patients [68]. However, further investigations focused on hemodialysis patients have yielded contradicting results [71,73]. The association of TRAIL with CV mortality in those patients was reported as non-significant [68,71,73], but interestingly, data from a proteomic study identified TRAIL-R2 as a protein associated with CV mortality in hemodialysis patients [79]. One thing that we must keep in mind when analyzing these studies is that patients with kidney failure have a unique risk factor profile and do not reflect the standard population.

Apart from predicting the outcome and therefore serving as a risk marker, TRAIL is considered to participate in the pathogenesis of various CVDs, including heart failure, atherosclerosis, coronary artery disease, atrial fibrillation (AF), and stroke. The discussion is ongoing about whether TRAIL and its death receptors act also as risk factors. Below, we summarize the knowledge on selected topics.

### 4.1. Heart Failure

Higher levels of TRAIL have been associated with better prognosis in HF patients both with reduced ejection fraction [66] and preserved ejection fraction [74]. Low levels of TRAIL in patients with HF were found to be an independent predictor of the risk of rehospitalization and death (in a 16-month follow-up study) [66]. The risk of death was confirmed in the same population in an extended follow-up of five years [72]. Data from animal models show that mechanical stretch (from mechanical overload) activated death receptor-mediated apoptotic signaling in cardiomyocytes [87]. These results suggest that activation of the TRAIL pathway has been implicated in the development and progression of heart failure, but the mechanism of cardioprotection exerted by TRAIL has not been yet fully elucidated. One suggestion is that higher levels of TRAIL possibly reflects the need for TRAIL-induced apoptosis to resolve inflammation [74], but data from an animal model actually suggests the opposite. The injection of recombinant TRAIL significantly reduced cardiac fibrosis and apoptosis and, as consequence, prevented more relevant cardiac structural changes in a mouse model of cardiomyopathy [13]. In this study, it was suggested that, contrary to its pro-apoptotic effects, it may be the result of triggering non-apoptotic signals in normal cells (promoting survival, migration and proliferation of primary vascular smooth muscles cells) [10,83]. However, in patients with Chagas cardiomyopathy serum, TRAIL levels were enhanced, correlated with decreased left ventricular ejection fraction and left ventricular diastolic dimension [23]. Similar results come from a study of men with nonischemic dilated cardiomyopathy, where the TRAIL concentration was also higher compared to the control group [22]. The prognostic value of these results and their agreement with data from HF patients is unknown.

### 4.2. Coronary Artery Disease

Emerging evidence has also demonstrated that TRAIL is involved in atherosclerosis development. However, the role of TRAIL in this process is equivocal. One study showed that TRAIL induces apoptosis and has pro-inflammatory effects in human endothelial cells [88]. On the other hand, several studies demonstrated possible anti-atherogenic and anti-inflammatory activity both in vitro and in vivo [15,81]. Cross-sectional and prospective human studies are also in agreement with this hypothesis [29,33,34]. Although we must remember that a cause-and-effect relationship cannot be concluded from observational studies, taking into consideration results from in vitro studies, there seems to be enough evidence to support the vasoprotective role of TRAIL. Potentially, since both endothelial cells and vascular smooth muscle cells express TRAIL receptors, TRAIL may be a molecule that can promote cell survival by activating intracellular signaling pathways, such as ERK/MAPK, Akt and NF-κB, which are known to promote survival and proliferation [10,82].

Few studies have focused on diabetic patients. Metabolic factors are involved in the vascular injury that accelerates the development of atherosclerosis [89]; therefore, there is a higher incidence of CVDs in diabetic patients. A hypothesis has been formed that the higher incidence of CVDs in diabetic patients is related to increased death-receptor activated apoptosis in the CV system in response to metabolic stress [75]. Kawano et al. tried to examine the association of TRAIL with atherosclerosis (the surrogate marker measured was intima-media thickness) in patients with type 2 diabetes but without any symptoms of coronary artery disease and HF [29]. The study showed the correlation between TRAIL and intima-media thickness only in a subset of diabetic patients with macrovascular disease where TRAIL was significantly and inversely correlated with carotid intima-media thickness. The authors concluded that TRAIL might not be helpful in the detection of early-stage atherosclerotic lesions.

Several studies showed that the levels of TRAIL are decreased in patients with coronary artery disease but with different significance. A cross-sectional study in patients undergoing angiography reported a borderline reduction of TRAIL levels compared to healthy controls [32]. Yet, another study found that circulating TRAIL was substantially lower in patients with acute coronary syndrome than those with stable angina and healthy subjects [56]. These differences in the impact of TRAIL might be explained by another study, which found that TRAIL can be a potential marker of the severity of coronary artery disease. Serum TRAIL levels were not only significantly lower in patients with coronary artery disease but also inversely associated with the number of diseased vessels independent of other coronary risk factors [34]. Secchiero et al. reported that in the acute phase of myocardial infarction serum concentration of TRAIL was acutely decreased and tended to increase in following days [67]. After 6 to 12 months of follow-up, its level was not significantly different from those of healthy control subjects. TRAIL was also inversely correlated with biochemical markers of myocardial injury (creatinine kinase and creatinine kinase-MB fraction) and cardiac dysfunction (B-type natriuretic peptide). Moreover, TRAIL predicted not only in-hospital and long-term mortality (follow-up of 12 months) but also the incidence of heart failure [67]. Thus, low TRAIL levels at discharge represent a possible predictor of future CV events following acute myocardial infarction.

Although myocardial infarction is a known cause of the release of apoptotic biomarkers, explanations for low levels of TRAIL in acute coronary syndrome are inconsistent. The most commonly proposed reason is its consumption of the atherosclerotic plaques. Other reasons may include the simultaneous increase in OPG, which is a decoy receptor and may bind TRAIL in the acute phase of myocardial infarction and increase in metalloproteinase-2, which is also released following acute myocardial infarction and is responsible for inducing TRAIL cleavage [62]. Additionally, transcription factor Egr-1, upregulated during atherosclerosis, is known to downregulate TRAIL expression in endothelial cells [90]. Thus, the hypotheses about TRAIL consumption into the plaques or reduction in its production need to be further pursued and clarified.

### 4.3. Atrial Fibrillation

AF promotes tissue fibrosis [91], which is an important contributor of AF recurrences, resistance to therapy, and complications [92]. Cardiac remodeling is considered a reparative process of replacing degenerative or dead myocytes. Atrial fibrosis involves processes of necrosis and apoptosis, hence its pathophysiological link to TRAIL. In agreement with this are reports that the restoration of sinus rhythm in patients with AF is associated with a decrease in the serum concentration of TRAIL [24,27]. On the other hand, published studies did not confirm the prognostic utility of TRAIL in the prediction of AF occurrence in patients with sinus rhythm [26] and the restoration of sinus rhythm in patients with AF treated pharmacologically [27] or with electrical cardioversion [25]. Deftereos et al. reported that TRAIL concentration shows transcardiac gradient (coronary sinus concentration minus aortic root concentration) in patients with AF after electrical cardioversion and in their half-year follow-up transcardiac gradient, but not TRAIL concentration alone, was inversely correlated with recurrence of AF [25]. Based on these results, the hypothesis of cardiac TRAIL production was made. Proteomic studies have also identified TRAIL-R2 as a risk factor for AF [28].

### 4.4. Cerebral Ischemia

Since the involvement of TRAIL in atherosclerosis was proven, further studies on its relevance in cerebrovascular diseases were conducted. The abundance of studies on the involvement of TRAIL in atherosclerosis has led to further research on its relevance in cerebrovascular disorders. TRAIL detection in cases of brain ischemia can be explained not only by atherosclerosis; TRAIL in the brain can be released by glia [93], injured neurons, and also circulating leukocytes [94]. Moreover, data from animal models showed TRAIL expression in ischemic areas of the brain, suggesting its potency in inducing death in neurons [16]. Kang et al. analyzed the serum levels of TRAIL in patients with acute ischemic stroke. In these patients, low levels of TRAIL were associated with ischemic stroke severity and stroke volume assessed by imaging [45]. This is in agreement with another study where TRAIL concentration in patients with large-artery atherosclerotic stroke was significantly lower compared to healthy controls. In a three-month follow-up, levels of TRAIL were found to be negatively correlated with prognosis measured by the modified Rankin Scale [46]. It is suggested from in vivo studies that blocking the TRAIL apoptosis pathway may be effective in preventing neuronal death in patients with stroke [95].

## 5. Conclusions

Considering all of these data together, the TRAIL pathway system undoubtedly has gathered attention as a player in increased CV risk. Its role has been investigated in various types of CVDs. The evidence from most of them associate reduced TRAIL and increased TRAIL-R2 concentration with many forms of heart disorders. It is, however, unclear whether low TRAIL levels should be considered as a risk factor rather than a risk marker of CVD. Understanding the association between CVD and TRAIL has important implications for disease management such as targeted therapies or prognosis. Since human-recombinant TRAIL is being tested in cancer studies, it is possible to apply this therapy to another field. To do so, further work is needed to better define the role of TRAIL in CVDs.

## Figures and Tables

**Table 1:** Original Studies Assessing the Relationship between TRAIL and TRAIL-R2 and Cardiovascular Risk.

Author	Year	Design of the Study	Population	Number of Patients	Parameters Assessed	Results and Key Observations
**Heart Failure**						
Osmancik et al. [20]	2013	Prospective observational study	Patients with heart failure treated with CRT	81	Six-month evaluation and two-year follow-up after implantation	TRAIL did not differ in responders and nonresponders, TRAIL did not predict mortality
Stenemo et al. [21]	2017	Prospective observational study	Elderly patients without heart failure at baseline	1586	Eight-year and 10-year follow-up/proteomics profilling	TRAIL-R2 associated with incident heart failure at follow-up and worsened LV systolic function at baseline
**Cardiomyopathy**						
Schoppet et al. [22]	2005	Case-control study	Man with nonischemic dilated cardiomyopathy	105 and 86 controls	Comparison to control group	TRAIL elevated comparing to controls, correlating with LV end-diastolic diameter
Lula et al. [23]	2009	Case-control study	Patients with Chagas cardiomyopathy	31 and 15 controls	TRAIL concentration compared between groups and hemodynamic parameters	TRAIL concentration enhanced and correlate with LV ejection fraction and LV diastolic diameter
**Atrial Fibrillation (AF)**						
Osmancik et al. [24]	2010	Prospective observational study	Patients with AF	25	Intervention-ablation	TRAIL concentration decreased in patients successfully ablated
Deftereos et al. [25]	2012	Prospective observational study	Patients with persisted AF, successfully cardioverted to sinus rhythm	45	Six-month follow-up, measured transcardiac gradients (coronary sinus concentration minus aortic root concentration)	TRAIL concentration without differences in group with and without AF recurrence, TRAIL transcardiac gradient was negative predictor of AF recurrence
Muller et al. [26]	2013	Prospective observational study	Patients with sinus rhythm, without history of AF, undergoing cardiac surgery	33	TRAIL concentration measured pre- and postoperatively	TRAIL did not predict postoperative AF
Rewiuk et al. [27]	2015	Prospective observational study	Patients with acute onset of AF	60	TRAIL concentration measured initially and 7 to 10 days after pharmacological cardioversion	TRAIL did not predict restoration of sinus rhythm, increase in TRAIL concentration in sinus rhythm maintenance group
Chua et al. [28]	2019	Prospective observational study	Patients with known AF	638	Logistic regression with machine learning algorithms to determine AF risk factors	TRAIL-R2 associated with AF
**Atherosclerosis**						
Kawano et al. [29]	2011	Cross-sectional study	Patients with type 2 diabetes without symptoms of CAD	416	TRAIL concentration measured in correlation with atherosclerotic lesion (IMT)	TRAIL did not correlate with IMT and not differ in group with calcified plaque and without
Deftereos et al. [30]	2011	Cross-sectional study	Patients with stable angina or positive ischemia noninvasive test	56	TRAIL concentration measured during left cardiac catherization; IVUS plaque assessment	TRAIL associated with histologic prototype of plaque
Arcidiacono et al. [31]	2018	Prospective observational study	Patients with chronic kidney disease without previous CV events	378	TRAIL concentration compared with appearance of plaque baseline and after 24-month of follow-up	TRAIL associated negatively with plaque at baseline and with new atheromatous plaques after 24 months
**Coronary Artery Disease**						
Schoppet et al. [32]	2005	Cross-sectional study	Men undergoing coronary angiography for suspected CAD	363	TRAIL concentration compared between group with and without CAD	TRAIL and TRAIL/OPG ratio not correlated with presence or severity of CAD
Satoh et al. [33]	2009	Prospective observational study	Patients with CAD treated with percutaneous coronary intervention	85 and 50 controls	0.5-year follow-up	TRAIL concentration higher in CAD patients and non-restenosis group
Mori et al. [34]	2010	Cross-sectional study	Patients undergoing coronary angiography	285	TRAIL concentration assessed with CAD severity	TRAIL concentration was inversely correlated with severity of CAD
Secchiero et al. [35]	2010	Case-control study	Patients with AMI	113 and 21 with unstable angina and 120 controls	TRAIL concentration compared between groups	TRAIL concentration decreased in AMI patients, CAD patients characterized by an increased OPG/TRAIL ratio
Shaker et al. [36]	2010	Case-control study	Male patients with AMI and CAD	28 with AMI, 30 with CAD and 20 controls	TRAIL concentration compared between groups	TRAIL concentration lover in AMI patients
Deftereos et al. [37]	2012	Prospective observational study	Patients undergoing percutaneous coronary intervention with drug-eluting stent	67	12-months follow-up	TRAIL negatively correlated indices of neointimal hyperplasia and positively correlated in-stent minimum lumen area
Song et al. [38]	2012	Case-control study	Patients with CAD undergoing stent implantation	42 and 17 controls	0.5-year follow-up	TRAIL concentration was increased 1 month after angioplasty
Luz et al. [39]	2016	Prospective observational study	Patients with STEMI	66	TRAIL concentration measured between group treated with postconditioning and without, nine-month follow-up	TRAIL was augmented by postconditioning and correlated to infarct size and LVEF
Manuneedhi Cholan et al. [40]	2018	Case-control study	Patients with stable CAD	9 and 7 controls	TRAIL and F2-isoprostanes concentration compared between groups	TRAIL concentration was reduced in CAD patients and correlated with marker of oxidative stress
Teringova et al. [41]	2018	Prospective observational study	Patients with STEMI treated with primary percutaneous coronary intervention	101	TRAIL concentration measured at baseline and one month after, two-year follow-up	TRAIL reaches its lowest serum concentration after reperfusion, low TRAIL concentration is associated with worse LVEF
**Vascular Calcification**						
Chasseraud et al. [42]	2011	Cross-sectional study	Hemodialysis patients	38	TRAIL concentration measured in hemodialysis patients and compared with calcification levels	TRAIL decreased in serum of hemodialysis patients but not correlate with calcification
Moon et al. [43]	2019	Cross-sectional study	Patients with diabetes and without diabetes	71	Diagnosis of PAD based on ABI results; calcification determined by computed tomography scan	TRAIL was downregulated in patient with PAD and vascular calcification
**Cerebrovascular Disease**						
Sarchielli et al. [44]	2013	Case-control study	Patients with silent brain infarction and lacunar infarct	49 and 31 controls	Assessment of pathophysiology of silent brain infarction	TRAIL concentration was higher in patients with silent brain infarction
Kang et al. [45]	2015	Cross-sectional study	Patients with ischemic acute stroke	293	Assessment of TRAIL correlation with stroke volume	Low concentration of TRAIL correlated with stroke severity
Pan et al. [46]	2015	Prospective observational study	Patients with large artery atherosclerosis stroke	132 and 60 controls	Three-month follow-up	TRAIL concentration lower in patients with large artery atherosclerosis stroke; TRAIL negatively correlated with prognosis
**Other**						
O’Sullivan et al. [47]	2010	Case-control study	Patients with PAD	83 and 21 controls	PAD assessed using ABI, TRAIL concentration measured between groups with PAD and diabetes	TRAIL concentration was higher in patients with PAD
Karatolios et al. [48]	2011	Cross-sectional study	Patients with pericardial effusion	83	Assessment TRAIL concentration in pericardial effusion associated with malignancy, CAD and non-malignant	TRAIL concentration was higher in pericardial effusion associated with malignancy and CAD
Zhou et al. [49]	2014	Validation study	Pregnant women	812	Serum samples taken from 8 to 20 week gestation; than assessed those who developed hypertension	TRAIL concentration lower in patients who developed pregnancy hypertension than uncomplicated pregnancies
Liu et al. [50]	2015	Prospective observational study	Patients with pulmonary hypertension	78 and 80 controls	Comparison of TRAIL concentration between groups, 2-years follow-up	TRAIL concentration higher in patients with pulmonary hypertension; elevated TRAIL associated with eventual complications
**General Cardiovascular Risk**						
Wigren et al. [51]	2018	Cross-sectional study	Patients with systemic lupus erythematosus	484 and 253 controls	Comparison of TRAIL-R2 concentration between groups	14% of patients had CVD (CAD, cerebrovascular disease, PAD); patients with CVD had higher concentration of TRAIL-R2 than those without
Goncalves et al. [52]	2019	Prospective observational study	CPIP cohort (patients with atherosclerosis)	558	37-month follow-up	Higher TRAIL-R2 associated with higher CV risk in future

Abbreviations: ABI—ankle-brachial index, AF—atrial fibrillation, AMI—acute myocardial infarction, CAD—coronary artery disease, CRT—cardiac resynchronization therapy, CV—cardiovascular, IMT—intima-media thickness, IVUS—intravascular ultrasound, LV—left ventricle, LVEF—left ventricular ejection fraction, OPG—osteoprotegerin, PAD—peripheral artery disease, STEMI—ST elevation myocardial infarction TRAIL—TNF-related apoptosis-inducing ligand, TRAIL-R2—TNF-related apoptosis-inducing ligand receptor 2.

**Table 2:** In Vitro Studies on Human Samples Assessing Role of TRAIL and TRAIL Receptors in Cardiovascular Diseases.

Author	Year	Investigated Disease	Population	Number of Patients	Parameters Assessed	Results and Key Observations
Yndestad et al. [53]	2002	Heart failure	Patients with heart failure	8 and 8 controls	Analysis of gene expression in peripheral blood mononuclear cells	TRAIL gene downregulated in chronic heart failure patients
Cao et al. [54]	2011	Atrial fibrillation	Patients with AF	48 and 48 controls	Tissue obtained during mitral valve surgery	TRAIL gene expression upregulated
Schoppet et al. [55]	2004	Atherosclerosis	Patients’ samples of vascular tissue	8 and 4 normal vessels	Analysis of samples of Mönckeberg’s sclerosis and atherosclerotic arteries	TRAIL detected in calcified regions of Mönckeberg’s sclerosis and atherosclerotic arteries
Michowitz et al. [56]	2005	Atherosclerosis	Patient’s samples of atherosclerotic plaques	24	TRAIL expression assessed on peripheral mononuclear cells when stimulated by oxLDL	TRAIL expression enhanced by oxLDL in atherosclerotic lesions; TRAIL serum concentration reduced in patients with unstable CAD
Niessner et al. [57]	2006	Atherosclerosis	Patients with ACS	31	TRAIL expression assessed on CD4 T-cells when stimulated by IFNα produced by activated plasmacytoid dendritic cells	TRAIL expression is enhanced by IFNα in atherosclerotic lesions
Goncalvez et al.	2019	Atherosclerosis	Patients’ samples of carotid plaques	202	TRAIL expression analyzed in atherosclerotic lesion	TRAIL-R2 and TRAIL expression were increased in symptomatic carotid plaques
Nakajima et al. [58]	2003	Coronary artery disease	Patients with AMI	26 and 16 controls	Compared expression of TRAIL on peripheral blood mononuclear cells between groups	TRAIL expression was enhanced in AMI patients
Sato et al. [59]	2006	Coronary artery disease	Patients with ACS	50 and 33 controls	Examined whether the TRAIL pathway was involved in CD4 T cell-mediated apoptosis	TRAIL expression enhanced on CD4 T-cells in patients with ACS
Corallini et al. [60]	2009	Coronary artery disease	Patients with AMI	80 and 40 controls	Analysed the relationship of TRAIL with mesenchymal stem cells (role in regeneration after AMI)	TNFα enhanced the migration of mesenchymal stem cells in response to TRAIL
Sato et al. [61]	2010	Coronary artery disease	Patients with ACS	55 and 34 controls	Intervention-administration of statins and TRAIL-specific antibodies	TRAIL-R2 upregulated on endothelial cells, T cell mediated endothelial death was dependent on the TRAIL pathway
Secchiero et al. [62]	2010	Coronary artery disease	Patients with AMI	30	Evaluated the potential role of metalloproteinase 2 in promoting the cleavage of TRAIL after AMI	TRAIL concentration showed inverse correlation with MMP2/TIMP2 ratio
Liu et al. [63]	2007	Vascular calcification	Patients’ aortic samples taken during abdominal aortic aneurysm	33 and 8 controls	calcification levels were determined by computed tomography scan	TRAIL and TRAIL-R1 expression were higher in aneurysm samples and in calcified samples
Galeone et al. [64]	2013	Vascular calcification	Patients’ samples of severe calcific aortic stenosis taken during valve replacement	10 and 10 controls	Immunohistochemistry and confocal microscopy used for TRAIL immunostaining	higher TRAIL concentration detected in calcific aortic valves and serum
Hameed et al. [17]	2012	Pulmonary hypertension	Specimens of human pulmonary artery smooth muscle cells	-	Assessment of TRAIL expression in smooth muscle cells	TRAIL upregulated in pulmonary artery smooth muscle cells in patients with pulmonary hypertension
Tisato et al. [65]	2013	Chronic venous disease	Specimens of venous endothelial cells from patients with chronic venous disease	20	Assessment of TRAIL expression in correlation with hemodynamic parameters and after GM-CSF exposure	TRAIL expression correlated positively with resistance index and GM-CSF

Abbreviations: AF—atrial fibrillation, AMI—acute myocardial infarction, ACS—acute coronary syndrome, CAD—coronary artery disease, GM-CSF—granulocyte-macrophage colony-stimulating factor, IFNα—interferon α, MMP2/TIMP2—metalloproteinase-2/tissue inhibitor molecule 2, oxLDL—oxidized low-density lipoprotein, TNFα—tumor necrosis factor α, TRAIL—TNF-related apoptosis-inducing ligand, TRAIL-R1—TNF-related apoptosis-inducing ligand receptor 1, TRAIL-R2—TNF-related apoptosis-inducing ligand receptor 2.

**Table 3:** Summary of the Studies Assessing the Association between TRAIL or TRAIL-R2 with Cardiovascular Patient-Related Outcome Measures.

Author	Year	Population	Measured Protein	Number of Patients	Follow-Up	Death/CV Deaths	Assessed Outcome					Relation TRAIL to All-Cause Mortality	Relation TRAIL to CV Mortality	Comments
							MI	Stroke	CV Death	All-Cause Death	Re-Hospitalization			
Niessner et al. [66]	2009	Patients with HF *	TRAIL	351	16 months	93/ND	-	-	-	yes	yes	inversely *p* < 0.001	n/a	TRAIL predicted outcome (all-cause mortality and rehospitalization)
Secchiero et al. [67]	2009	Patients with AMI	TRAIL	60 and 60 control group	12 months	10/9	-	-	yes	yes	-	ND	inversely *p* = 0.001	TRAIL concentration decreased at baseline, low levels of TRAIL at discharge was prognostic factor of cardiac death and heart failure at 12 months
Liabeuf et al. [68]	2010	Chronic kidney disease patients	TRAIL	130	2 years	36/19	-	-	yes	yes	-	inversely *p* = 0.010	NSS	lowest TRAIL was associated with infectious but not CV mortality
Volpato et al. [69]	2011	inCHIANTI study (older people with CVD)	TRAIL	1282	6 years	259/112	-	-	yes	yes	-	inversely *p* = 0.008	inversely ND	an association was found between prevalent CV disease and TRAIL
Osmancik et al. [70]	2013	Patients with ACS	TRAIL	295	0.5 year	12/ND	yes	yes (but not evaluated)	-	yes	yes	inversely *p* = 0.001	n/a	TRAIL predicted all-cause mortality, re-MI and combined end point (death and hospitalization for HF)
Mori et al. [71]	2013	Male hemodialysis patients	TRAIL	149	36 months	33/11	-	-	yes	yes	-	inversely *p* = 0.011	NSS	TRAIL associated with infectious and all-cause mortality but not CV mortality
Richter et al. [72]	2013	Patients with HF *	TRAIL	349	5 years	195/145	-	-	-	yes	-	inversely *p* < 0.001	n/a	TRAIL predicted all-cause mortality
Kuzniewski et al. [73]	2016	Hemodialysis patients	TRAIL	69 and 35 controls	7 years	39/31	-	-	yes	yes	-	NSS	NSS	TRAIL did not predict CV mortality; OPG/TRAIL ratio positively predicted all-cause and CV mortality
Hage et al. [74]	2017	Patients with HF with preserved ejection fraction/proteomic study	TRAIL and TRAIL-R2	86	1.5 years	11/ND	-	-	-	yes	yes	ND (composite outcome)	n/a	TRAIL and TRAIL-R2 predicting outcome (all-cause mortality or re-hospitalization
Mattisson et al. [75]	2017	MDC study (general population)	TRAIL-R2	4742	19 years	ND/278	yes	yes	yes	-	-	n/a	n/a	higher TRAIL-R2 was associated with increased risk of CV events (myocardial infarction and stroke)
Skau et al. [76]	2017	Patients with AMI	TRAIL-R2	847	7 years	207/ND	-	-	-	yes	-	n/a	n/a	TRAIL-R2 predicted all-cause mortality
Ajala et al. [77]	2018	Smokers	TRAIL	474	8 years	83/ND	-	-	-	yes	-	inversely *p* = 0.004	n/a	TRAIL concentration reduced in smokers with comorbid emphysema and CAD; related to reduced survival/ CAD assessed by quantifying coronary artery calcium
Nowak et al. [78]	2018	Patients with diabetes	TRAIL-R2	1211	6 years	ND	yes	yes	-	yes	-	n/a	n/a	TRAIL-R2 increased concentration associated with incident major adverse CV events
Feldreich et al. [79]	2019	MIMICK study (hemodialysis patients)/proteomic study	TRAIL-R2	183	43 months	ND/45	-	-	yes	-	-	n/a	n/a	TRAIL-R2 associated with CV mortality
Ferreira et al. [80]	2020	Patients with diabetes after MI/proteomic study	TRAIL-R2	5131	1.5 year	302/226	yes	yes	yes	yes	yes	n/a	n/a	TRAIL-R2 prognose all-cause mortality and CV death or HF hospitalization

* the same population. Abbreviations: ACS—acute coronary syndrome, AMI—acute myocardial infarction, CV—cardiovascular, CVD—cardiovascular disease, HF—heart failure, MI—myocardial infarction, ND—no data, NSS—not statistically significant.

## Data Availability

No new data were created or analyzed in this study. Data sharing is not applicable to this article.
